# Tirbanibulin 1% Ointment for the Treatment of Hyperkeratotic Actinic Keratosis

**DOI:** 10.1111/1346-8138.17845

**Published:** 2025-07-02

**Authors:** Federica Li Pomi, Andrea d'Aloja, Michelangelo Rottura, Mario Vaccaro, Francesco Borgia

**Affiliations:** ^1^ Department of Precision Medicine in Medical, Surgical and Critical Care (Me.Pre.C.C.) University of Palermo Palermo Italy; ^2^ Section of Dermatology, Department of Clinical and Experimental Medicine University of Messina Messina Italy; ^3^ Section of Pharmacology, Department of Clinical and Experimental Medicine University of Messina Messina Italy

**Keywords:** actinic keratosis, hyperkeratotic actinic keratosis, Olsen grade, tirbanibulin, treatment

## Abstract

Actinic keratosis (AK) is a common cutaneous keratinocyte dysplasia resulting from chronic ultraviolet (UV) exposure, typically presenting as erythematous, scaly patches or plaques on sun‐damaged skin. AK is recognized as the main precursor of cutaneous squamous cell carcinoma (cSCC). Given the unpredictable potential for progression, current guidelines recommend treating all AKs, irrespective of their clinical grade. However, many approved treatments are not indicated for hyperkeratotic AKs. Among topical therapies, tirbanibulin 1% ointment is a novel synthetic anti‐proliferative agent that has shown good results in treating non‐hyperkeratotic AKs on the face and scalp, both in clinical trials and real‐life experiences. However, its effectiveness in managing hyperkeratotic AKs remains unexplored. A retrospective, single‐center study was conducted at the Dermatology Unit of the University of Messina, Italy, between September 2024 and January 2025. The study included 51 hyperkeratotic AK lesions in 32 consecutive patients, treated with tirbanibulin ointment for five consecutive days. At the 2‐month follow‐up, total clearance (complete lesion resolution) was observed in 54.9% of hyperkeratotic lesions, whereas partial clearance (> 75% lesion reduction) was recorded in 25.5%. The treatment demonstrated a good safety profile, with high patient tolerability. LSRs were observed in 69.8% of patients (*n* = 22), of which 54.5% (*n* = 12/22) were classified as moderate, and 45.5% (*n* = 10/22) as mild. No patient discontinued the treatment due to the onset of adverse events. Our real‐world experience suggests the effectiveness and safety of tirbanibulin ointment for the treatment of hyperkeratotic AKs.

## 1 Introduction

Actinic keratosis (AK) is a common cutaneous keratinocyte dysplasia characterized by the abnormal proliferation of atypical epidermal keratinocytes, primarily caused by chronic ultraviolet (UV) exposure [1]. In the World Health Organization (WHO) classification of skin tumors, AK is listed as a precursor of cutaneous squamous cell carcinoma (cSCC), with the risk of progression for individual lesions estimated at 0.025%–16% annually [2, 3]. AKs typically present as rough, erythematous scaling patches or plaques, commonly arising in chronically sun‐damaged body areas, such as the face and scalp [1]. To standardize clinical evaluation, Olsen et al. proposed a classification system based on lesion thickness [4]. Grade 1 lesions are slightly palpable and better detected by touch than by sight. Grade 2 lesions are both visible and tactile, showing moderate thickness. Grade 3 lesions, or hyperkeratotic AKs, are characterized by pronounced keratinization. Current guidelines recommend treating all AKs, regardless of clinical grade, due to the unpredictable nature of lesion progression [1]. However, many approved AK treatments are not indicated for Olsen grade 3 patients. Non‐hyperkeratotic AKs (grades 1 and 2) are commonly included in clinical trials for AK therapies, whereas hyperkeratotic lesions (grade 3) are often excluded as hyperkeratosis limits penetration of topical treatments. Consequently, Olsen classification significantly influences the patient populations for which AK therapies are ultimately approved, with limited evidence of their efficacy in hyperkeratotic AKs [5]. Among topical therapies, tirbanibulin 1% ointment has shown good results in the treatment of non‐hyperkeratotic facial and scalp AKs, both in clinical trials and real‐life experiences, due to its dual anti‐proliferative and pro‐apoptotic mechanisms [6, 7]. Its efficacy in managing hypertrophic AKs remains still unexplored.

In order to address this gap, we performed a retrospective study to provide insights into tirbanibulin's therapeutic potential and expand its applicability in AK treatment.

## 2 Materials and Methods

### 2.1 Inclusion and Exclusion Criteria (Study Population)

A retrospective observational non‐randomized study was carried out at the Dermatology Unit of the University of Messina, Italy, between September 2024 and January 2025.

Adult patients diagnosed with clinically typical, visible, discrete, indolent hyperkeratotic AKs of the face or scalp in the context of the cancerization field were included.

Exclusion criteria included the presence of atypical, recalcitrant, painful, or rapidly evolving AKs, as well as clinically suspected skin cancers near the treatment area. Additional exclusion criteria were prior topical therapies for AKs within the previous 12 weeks.

The procedures followed here were in accordance with the ethical standards of the responsible committee on human experimentation and with the Helsinki Declaration of 1975, as revised in 1983. We have not used patients' names, initials, or hospital numbers. All patients had previously provided written informed consent for the use of their anonymized clinical data and to publish images, following institutional and ethical guidelines.

### 2.2 Objectives and Endpoints

The study aimed to evaluate the efficacy of tirbanibulin 1% ointment for the treatment of hyperkeratotic AKs.

The primary efficacy outcome was the percentage of lesions with clinical complete (100%) clearance at the 2‐month follow‐up, whereas the second outcome was the percentage of lesions with partial clearance, defined as a reduction of at least 75% of clinical and dermoscopic patterns.

The third objective was to assess the type and severity of local skin reactions (LSRs) and their potential correlation with patients' characteristics.

### 2.3 Study Design

#### 2.3.1 Baseline Evaluation

At baseline (T0), a physical examination of patients with the acquisition of clinical and dermoscopic photos of each hyperkeratotic AK lesion was performed. Clinically collected data included age, gender, phototype, personal history of skin cancer, and previous treatments for AKs (concluded at least 12 weeks before).

#### 2.3.2 Treatment Received by Patients

All patients received treatment with tirbanibulin 1% ointment once daily for five consecutive days. The ointment was to be applied to the affected hyperkeratotic AKs and cancerization field with a thin layer, avoiding application on open wounds or injured skin, as indicated in the package leaflet. All patients were informed in depth about the possible onset of LSRs.

Patients were instructed to record the exact timing of onset and the duration of LSRs, if present.

They were invited to come to the hospital personally or to send digital photos when they could not attend in person to allow physicians to evaluate LSRs. LSRs were defined as erythema, scaling, crusting, swelling, vesiculation, pustulation, and erosions. The assessment of LSRs was carried out with the use of a semiquantitative 4‐point scale with scores of 0—absent, 1—mild (slightly or barely perceptible), 2—moderate (a distinct presence), and 3—severe (marked or intense).

#### 2.3.3 Evaluation Post‐Treatment (T1)

Clinical and dermoscopic pictures were recorded 8 weeks (T1) after the start of the treatment to evaluate its efficacy. Efficacy was assessed for each AK as complete clearance (CC) (total disappearance of lesion) or partial clearance (PC) (> 75% reduction of lesion). CC and PC were considered mutually exclusive outcomes: lesions classified as having achieved CC were not included in the PC group. AKs that did not respond to treatment were clinically reassessed and, when deemed necessary, underwent biopsy for histological confirmation to rule out cSCC.

### 2.4 Statistical Analysis

A descriptive analysis was performed to assess the demographic and clinical characteristics of the enrolled patients, stratified by AK clearance. Additionally, a comparative analysis was conducted between patients with and without LSRs.

Due to a non‐normal distribution of some numerical variables, verified using the Kolmogorov–Smirnov test for normality, a non‐parametric approach was consistently adopted. Continuous variables were reported as medians with interquartile ranges (Q_1_–Q_3_), whereas categorical variables were presented as absolute and percentage frequencies.

The Mann–Whitney *U* test for independent samples and two‐tailed Pearson chi‐squared test were carried out to compare continuous variables and categorical variables, respectively.

A multivariate ordinal logistic regression model was used to identify potential predictors of treatment response, with age, gender, skin phototype, AK location (face or scalp), and prior treatments included as covariates. The onset of LSR was defined as the time interval between the initiation of drug application and the first appearance of a reaction, with the duration of LSR being recorded accordingly.

Spearman's rank correlation analysis was applied to identify associations between AK clearance and LSR occurrence. For each correlation, the Spearman's rank correlation coefficient (*r*_s_) was reported. Additionally, multivariate logistic regression models were applied to evaluate factors associated with LSR onset. Odds ratios (ORs) with 95% confidence intervals (CIs) were calculated for each covariate included in the multivariate models.

A *p*‐value < 0.05 was considered indicative of statistical significance. All analyses were performed using SPSS Statistics software, version 23.0 (IBM Corp., Armonk, NY, USA).

## 3 Results

### 3.1 Patient Characteristics

From September 2024 to January 2025, 32 patients (25 male, 7 female) with a median age of 78 years (Q_1_–Q_3_: 71–81) were enrolled in the study. Almost half of the patients had a type II skin phototype (*N* = 14; 43.8%), followed by type III (*N* = 13; 40.6%). A personal history of skin cancer was recorded in half of the population, including 7 patients (21.9%) with a prior diagnosis of cSCC, 7 (21.9%) with BCC, 1 (3.1%) with both cSCC and BCC, and 1 (3.1%) with melanoma.

A total of 51 AK was identified, of which 33 (64.7%) were located on the face and 18 (35.3%) on the scalp. Most patients (*N* = 20; 62.5%) had undergone previous treatments, including photodynamic therapy (PDT) (50%), cryotherapy (31.35%), and 5‐fluorouracil (5‐FU) 4% cream (21.9%).

Patients' characteristics are summarized in Table 1.

**TABLE 1:** Characteristics of the study population.

Median age (years)	78 (71–81)
Sex (%)	
Male	25 (78.1%)
Female	7 (21.9%)
Fitzpatrick skin type (%)	
I	1 (3.1)
II	14 (43.8)
III	13 (40.6)
IV	4 (12.5)
Hyperkeratotic AKs	51
Scalp	18 (35.3%)
Face	33 (64.7%)
Previous treatments (%)	
PDT	6 (18.2%)
PDT, 5‐FU 4%	3 (9.1%)
PDT, 5‐FU 4%, cryotherapy	1 (3.1%)
PDT, 5‐FU 4%, cryotherapy	1 (3.1%)
PDT, cryotherapy	3 (9.1%)
PDT, cryotherapy, 5‐FU 4% cream	1 (3.1%)
PDT, IMI 3.75% cream, cryotherapy	1 (3.1%)
5‐FU 4% cream	1 (3.1%)
None	12 (37.5%)
Number of previous treatments median	1 (0–2)
History of skin cancer (%)	*N* = 32
Melanoma	1 (3.1)
cSCC	7 (21.9)
BCC	7 (21.9)
BCC. cSCC	1 (3.1)
No history	16 (50.0)

Abbreviations: 5‐FU, 5‐fluorouracil; AK, actinic keratosis; BCC, basal cell carcinoma; cSCC, cutaneous squamous cell carcinoma; IMI, imiquimod; PDT, photodynamic therapy.

### 3.2 Efficacy

**FIGURE 1:**
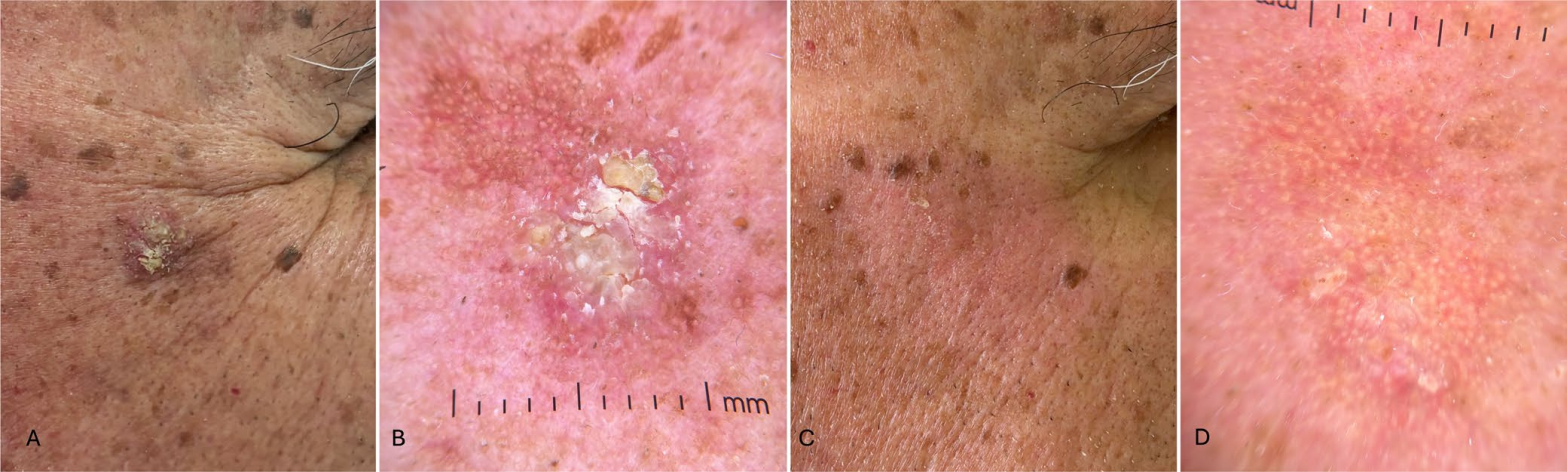
(A) AK in the right temporal area of a 72‐year‐old man; (B) dermoscopy (10×) highlights Olsen 3 AK, with scales and erythematous background; (C) clinical complete resolution of AK at 8‐week follow‐up; (D) reduction of the AK dermoscopic patterns, with slight persistence of perifollicular pseudo‐network.

**FIGURE 2:**
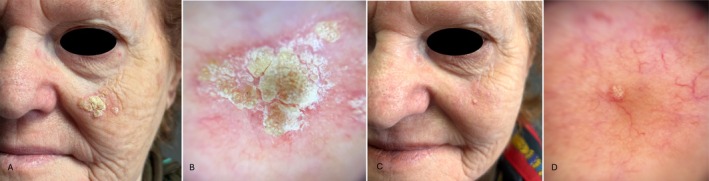
(A) AK in the left cheek in a 77‐year‐old woman; (B) dermoscopy (10×) highlights hyperkeratotic AK; (C) clinical almost complete resolution of AK at 8‐week follow‐up; (D) almost complete resolution of the AK dermoscopic patterns.

**FIGURE 3:**
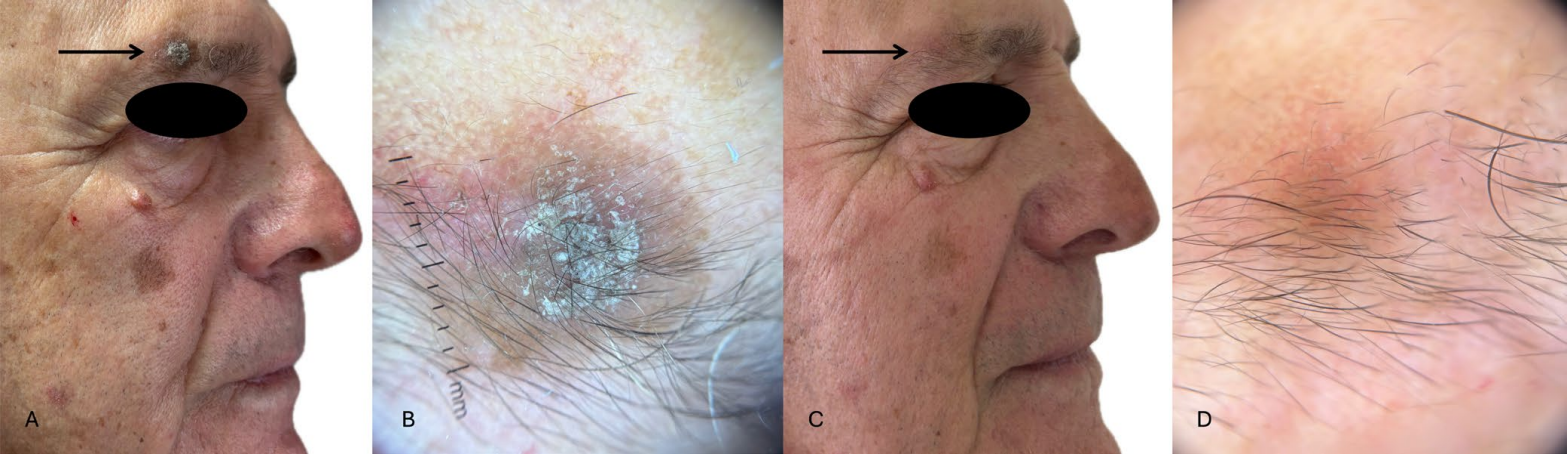
(A) AK in the right supraciliary area in a 78‐year‐old man; (B) dermoscopy (10×) highlights hyperkeratotic AK; (C) clinical complete resolution of AK at 8‐week follow‐up; (D) complete resolution of the AK dermoscopic patterns.

**FIGURE 4:**
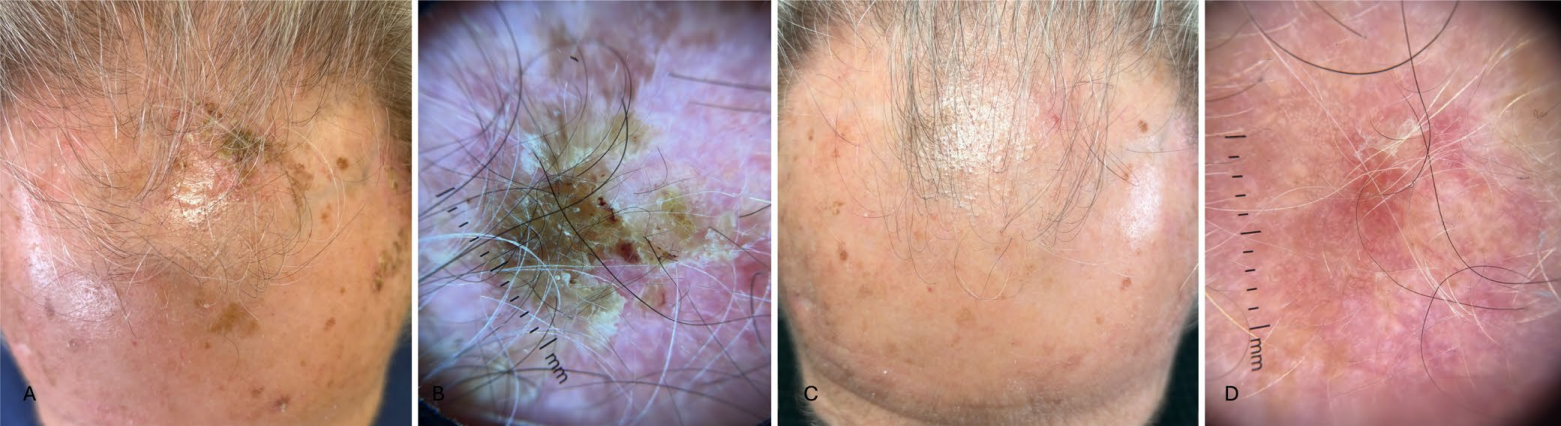
(A) AKs in the fronto‐parietal area of a 75‐year‐old man, in the context of the cancerization field; (B) dermoscopy (10×) highlights hyperkeratotic AK; (C) complete resolution of AKs at 8‐week follow‐up; (D) complete resolution of dermoscopic patterns.

At the 57‐day follow‐up (T1), CC was observed in 28/51 (54.9%) of lesions, while 13/51 (25.5%) showed PC, and 10/51 (19.6%) had no response to the treatment (Figures 1, 2, 3, 4) (Table 2). No statistically significant correlation was observed between the analyzed characteristics and AK clearance. Although not statistically significant, the ordinal logistic regression coefficients revealed a positive correlation between advanced age (coefficient: 0.05; 95% CI: −0.02/0.13), female gender (coefficient: 0.48; 95% CI: −1.18/2.13), lighter skin phototypes (types I and II) (coefficient: 0.46; 95% CI: −3.44/4.35), and AK location on the face (coefficient: 0.97; 95% c: −0.28/2.22) with AK clearance. In contrast, an inverse correlation was found in patients with a history of prior treatment (coefficient: −1.07; 95% CI: −2.40/0.26) (Table 3).

**TABLE 2:** Tirbanibulin's efficacy at the 8‐week follow‐up (T1).

	Complete response	Partial response	No response
AKs	28/51	13/51	10/51
Face	19/33	10/33	4/33
Scalp	9/18	3/18	6/18

Abbreviation: AK, actinic keratosis.

**TABLE 3:** Patient characteristics stratified by AK clearance (complete, partial, none) and multivariate ordinal logistic regression model for AK clearance.

	Complete clearance	Partial clearance	No response	Coefficient 95% CI	*p*
Median age (years)	75 (69–81)	81 (74–85)	77 (68–83)	0.05 (−0.02/0.13)	0.168
Sex (%); F					
Male	24 (85.7)	9 (69.2)	9 (90.0)	0.48 (−1.18/2.13)	0.572
Female	4 (14.3)	4 (30.8)	1 (10.0)		
Fitzpatrick skin type (%); I and II					
I	—	1 (7.7)	—	0.46 (−3.44/4.35)	0.818
II	9 (32.1)	5 (38.5)	6 (60.0)		
III	15 (53.6)	7 (53.8)	4 (40.0)		
IV	4 (14.3)	—			
AK location (%); face					
Scalp	9 (32.1)	3 (23.1)	6 (60.0)	0.97 (−0.28/2.22)	0.128
Face	19 (67.9)	10 (76.9)	4 (40.0)		
Previous treatments (%)					
None	10 (35.7)	4 (30.8)	2 (20.0)	−1.07 (−2.40/0.26)	0.113
Yes	18 (64.3)	9 (69.2)	8 (80.0)		

Abbreviations: AK, actinic keratosis; CI, confidence intervals.

**FIGURE 5:**
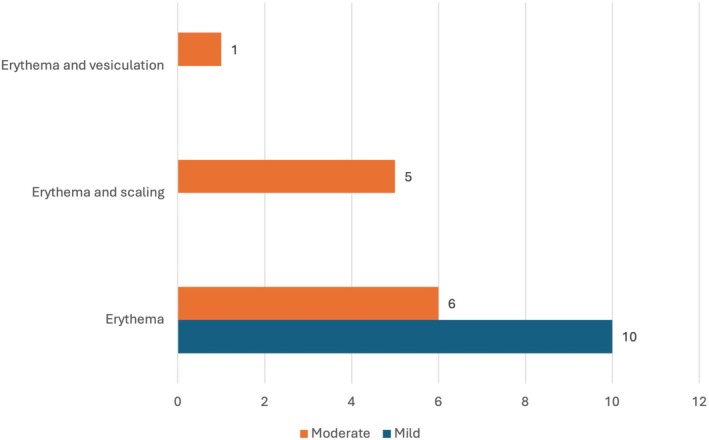
Local skin reactions stratified by severity.

### 3.3 Safety

Regarding tirbanibulin tolerability, 69.8% of patients (*n* = 22) developed LSRs, of which 54.5% (*n* = 12/22) were classified as moderate, while 45.5% (*n* = 10/22) as mild. No LSRs were classified as severe. No patient discontinued the treatment due to the onset of LSRs.

Erythema was the most frequent LSR, with five cases also exhibiting scaling and one case associated with vesiculation (Figure 5). The median time to onset of LSRs was 4 days (Q_1_–Q_3_: 3–4), with a median duration of 8 days (Q_1_–Q_3_: 7–10). The development of LSRs was positively correlated with improved clearance efficacy, as indicated by a Spearman correlation analysis, although the association was not statistically significant (*r*_s_ = 0.122; *p* = 0.395). No statistically significant associations were observed between patient characteristics and the occurrence of LSRs (Table 4).

**TABLE 4:** Sociodemographic and clinical characteristics of patients stratified by the presence of LSRs and factors associated with LSRs.

	With LSRs	Without LSRs	*p*	Multivariate OR, 95% CI	*p*
Median age (years)	77 (70–82)	79 (73–82)	0.263	0.96 (0.86–1.07)	0.456
Sex (%)					
Male	17 (77.3)	8 (80.0)	0.863	0.323 (0.04–3.00)	0.320
Female	5 (22.7)	2 (20.0)			
Fitzpatrick skin type (%)					
I and II	9 (40.9)	6 (60.0)	0.316	2.17 (0.47–9.95)	0.320
III and IV	13 (59.1)	4 (40.0)			
Previous treatments (%)					
None	7 (31.8)	5 (50.0)	0.325	3.72 (0.59–23.42)	0.161
Yes	15 (68.2)	5 (50.0)			

Abbreviations: LSRs, local skin reactions; OR, Odds ratios; CI, confidence intervals.

## 4 Discussion

AKs are dysplastic keratinocyte proliferations with potential for malignant transformation. Clinically, they appear as macules, papules, or hyperkeratotic plaques on an erythematous background, typically occurring in sun‐exposed areas of predisposed individuals over the age of 40 [8].

To standardize clinical evaluation, Olsen et al. classified AKs based on the overall thickness, with grade 1 AK being slightly palpable, grade 2 moderately thick, and grade 3 being very thick lesions [4].

Zalaudek et al. used dermoscopic examination to classify AK, with grade 1 AKs characterized by a red pseudo‐network pattern and white scales, grade 2 as an erythematous background with white to yellow keratotic areas and enlarged follicular openings (“strawberry pattern”), while grade 3 AKs display either enlarged follicular openings filled with keratotic plugs over a scaly, white‐yellow background or pronounced hyperkeratosis [9].

Conversely, Roewert‐Huber et al. histologically graded AKs by the depth of atypical keratinocytes in the epidermis. Grade AK I (mild) affects the lower third, grade AK II (moderate) extends to the lower two‐thirds, and grade AK III (severe) involves full‐thickness atypia, equivalent to SCC in situ [10].

However, emerging evidence has challenged the previously held assumption that the clinical grading of AKs directly correlates with histopathological findings [5].

Furthermore, other evidence highlighted that AK progression to invasive cSCC does not always adhere to the “classic pathway” of full‐thickness dysplasia. AKs can follow a “differentiated pathway” in which invasive cSCC may arise from the proliferation of atypical basaloid cells in the epidermal basal layer (i.e., AK I lesions) [11].

From these premises, it emerges the critical need to treat all AKs, irrespective of their clinical grade, along with addressing the broader cancerization field, as both clinical and histopathological assessments are necessary for comprehensive risk evaluation [12].

Various treatment options with different clearance rates and safety profiles are currently available for AK management [1].

AK therapeutical options can be divided into lesion‐directed and field‐directed therapies. “Lesion‐directed” therapies focus on single or a limited number of AK lesions within a specific area, whereas' field‐directed' herapies address the entire cancerization field [13]. Single or a few isolated AKs are commonly treated with cryotherapy or ablative lasers. In contrast, multiple lesions along with the surrounding photo‐damaged skin, namely the cancerization field, are generally treated using topical agents, including 4% 5‐FU cream, imiquimod (IMI) cream, diclofenac gel, 5‐FU plus salicylic acid (5‐FU/SA), or PDT with different application schedules [13]. The choice of treatment depends on the number, size, clinical grade, duration, and location of the lesions, as well as patient compliance and overall health status. In clinical practice, combination treatment modalities are often employed to improve clearance rates, particularly in hyperkeratotic AK clearance [14]. Field therapies, however, are frequently associated with the onset of LSRs and require long application regimens of weeks or months, potentially reducing patient adherence and treatment tolerability. Many approved therapies are primarily indicated for non‐hyperkeratotic AKs, as hyperkeratosis poses challenges in drug penetration [5]. Lesion curettage, ablative procedures (e.g., laser ablation), or the application of keratolytic agents (such as urea or salicylic acid) 2 weeks before treatment are commonly recommended to enhance drug absorption [1].

Recently, tirbanibulin ointment has shown good efficacy, excellent tolerability, and high compliance due to the short application regimen of five consecutive days. Tirbanibulin is a novel synthetic anti‐proliferative agent that inhibits tubulin polymerization—a structural protein critical for cell migration, protein transport, and cell division. It induces p53 expression, thus arresting the cell cycle at the growth 2 and mitosis (G2/M) interphase in proliferating cells. Furthermore, it activates intrinsic and extrinsic apoptotic signaling cascades through hyperphosphorylation of Bcl‐2, cleavage of caspases 8 and 9, and subsequent activation of caspase 3 and poly (ADP‐ribose) polymerase cleavage [15]. Additionally, it indirectly downregulates Src kinase signaling—upregulated in AKs and cSCC—by disrupting the microtubule network and interfering with cell signaling pathways [6].

The efficacy of tirbanibulin in the treatment of non‐hyperkeratotic AKs has been demonstrated in two pivotal phase 3 clinical trials, achieving CC in 44% and 54% of patients, respectively [16]. These results are supported by real‐life experiences, which highlighted similar results [17, 18, 19, 20].

Emerging evidence suggests it may also exhibit efficacy in treating hyperkeratotic AK lesions. A prospective single‐center study highlighted that among 27 grade 3 AK lesions treated with tirbanibulin, 8 achieved CC by day 57. Furthermore, a notable proportion of lesions demonstrated PC, underscoring the potential of tirbanibulin beyond its currently indicated scope [20].

Moreover, in a multicenter study, tirbanibulin reached 35.6% CC and 51.9% PC in the treatment of both Olsen 2/3 AKs, performing similarly to Olsen 1 AK lesions [21].

In our study, tirbanibulin reached 54.9% of CC and 28% of PC in 51 hyperkeratotic AKs on the face and scalp. Tirbanibulin appears to be more effective in treating facial AKs than scalp AKs, possibly due to differences in skin structure and drug absorption. Facial skin is thinner and more permeable, which may allow for better drug penetration into the epidermal layers where AKs develop. In contrast, the scalp has a thicker stratum corneum, which can act as a barrier, thus reducing tirbanibulin absorption, potentially limiting its efficacy.

Tirbanibulin has shown unique features in terms of mechanism of action, including its dual activity on microtubule dynamics and Src kinase signaling, short treatment duration, clinical outcomes, and favorable tolerability profile. These characteristics may support its use across various AK presentations, including hyperkeratotic forms, maintaining an excellent safety profile even when applied on a large field of up to 100 cm^2^ [22, 23].

Although recently approved, tirbanibulin is demonstrating great versatility in addressing various dermatological conditions, including viral lesions and neoplastic entities such as cSCC and BCC [24, 25, 26, 27, 28, 29]. Additionally, an emerging body of evidence suggests that tirbanibulin may have anti‐aging and skin‐lightening properties [30, 31]. Therefore, it is unsurprising that, even in this case, tirbanibulin can show efficacy for pathologies other than its initial approval, including hyperkeratotic AKs. In our study, no mechanical pre‐treatment (e.g., curettage or ablative techniques) or prior application of keratolytic agents was performed before starting the treatment with tirbanibulin ointment, to directly assess treatment efficacy without the confounding effect of lesion debridement. Nonetheless, the results have been encouraging, consistent with those observed in non‐hyperkeratotic lesions, confirming the good therapeutic efficacy previously observed in both clinical trials and real‐world studies.

Moreover, its short treatment schedule of 5 days improves patient compliance, particularly for those who struggle with prolonged regimens. Additionally, its favorable tolerability profile reduces the likelihood of severe LSRs, often associated with other treatments. In our cohort, no patient developed severe LSR, with mild erythema (*n* = 10) being the most frequent adverse event recorded.

Our study has some limitations, including the relatively small sample size and the lack of a control group, which prevents direct comparison with other treatment modalities. Moreover, the short follow‐up duration of 8 weeks is another constraint, as it does not allow for assessing long‐term efficacy or recurrence rates.

Lastly, the absence of histopathological confirmation of lesion clearance is a methodological limitation, as clinical evaluation alone may overestimate treatment success.

Future research should address these limitations through larger, randomized controlled trials with extended follow‐up periods to evaluate the durability of tirbanibulin's effects. Additionally, studies comparing tirbanibulin with other therapeutic approaches may yield insights into its antineoplastic effects and broaden its application scope.

## 5 Conclusion

This study contributes to the growing evidence supporting tirbanibulin's versatility in managing AKs. Although data on its use for hyperkeratotic lesions remain poor, these findings underscore the potential of tirbanibulin as a valuable addition to the poor therapeutic options for hyperkeratotic AK management. Given the significant public health burden of AKs and their role as precursors to cSCC, expanding the range of effective and patient‐friendly treatments remains a clinical priority.

## Disclosure

Institutional Review Board statement: The study was conducted in accordance with the Declaration of Helsinki of 1975, as revised in 1983. Patient names, initials, or hospital numbers have not been used.

## Consent

Informed consent was obtained from all subjects involved in the study.

## Conflicts of Interest

The authors declare no conflicts of interest.

## Data Availability

The data that support the findings of this study are available from the corresponding author upon reasonable request.

## References

[1] L. Kandolf , K. Peris , J. Malvehy , et al., “European Consensus‐Based Interdisciplinary Guideline for Diagnosis, Treatment and Prevention of Actinic Keratoses, Epithelial UV −Induced Dysplasia and Field Cancerization on Behalf of European Association of Dermato‐Oncology, European Dermatology Forum, European Academy of Dermatology and Venereology and Union of Medical Specialists (Union Européenne Des Médecins Spécialistes),” Journal of the European Academy of Dermatology and Venereology 38, no. 6 (2024): 1024–1047, 10.1111/jdv.19897. 38451047

[2] R. G. Glogau , “The Risk of Progression to Invasive Disease,” Journal of the American Academy of Dermatology 42, no. 1 (2000): S23–S24, 10.1067/mjd.2000.103339. 10607353

[3] World Health Organization , “WHO Classification of Tumours Online,” (2023).

[4] E. A. Olsen , M. Lisa Abernethy , C. Kulp‐Shorten , et al., “A Double‐Blind, Vehicle‐Controlled Study Evaluating Masoprocol Cream in the Treatment of Actinic Keratoses on the Head and Neck,” Journal of the American Academy of Dermatology 24, no. 5 (1991): 738–743, 10.1016/0190-9622(91)70113-G. 1869646

[5] L. Schmitz , P. Kahl , M. Majores , E. Bierhoff , E. Stockfleth , and T. Dirschka , “Actinic Keratosis: Correlation Between Clinical and Histological Classification Systems,” Journal of the European Academy of Dermatology and Venereology 30, no. 8 (2016): 1303–1307, 10.1111/jdv.13626. 26955898

[6] T. Schlesinger , E. Stockfleth , A. Grada , and B. Berman , “Tirbanibulin for Actinic Keratosis: Insights Into the Mechanism of Action,” Clinical, Cosmetic & Investigational Dermatology 15 (2022): 2495–2506, 10.2147/CCID.S374122. 36415541 PMC9675993

[7] M. V. Heppt , I. Dykukha , S. Graziadio , R. Salido‐Vallejo , M. Chapman‐Rounds , and M. Edwards , “Comparative Efficacy and Safety of Tirbanibulin for Actinic Keratosis of the Face and Scalp in Europe: A Systematic Review and Network Meta‐Analysis of Randomized Controlled Trials,” Journal of Clinical Medicine 11, no. 6 (2022): 1654, 10.3390/jcm11061654. 35329979 PMC8952421

[8] C. P. H. Reinehr and R. M. Bakos , “Actinic Keratoses: Review of Clinical, Dermoscopic, and Therapeutic Aspects,” Anais Brasileiros de Dermatologia 94, no. 6 (2019): 637–657, 10.1016/j.abd.2019.10.004. 31789244 PMC6939186

[9] I. Zalaudek , S. Piana , E. Moscarella , et al., “Morphologic Grading and Treatment of Facial Actinic Keratosis,” Clinics in Dermatology 32, no. 1 (2014): 80–87, 10.1016/j.clindermatol.2013.05.028. 24314380

[10] J. Röwert‐Huber , M. J. Patel , T. Forschner , et al., “Actinic Keratosis Is an Early In Situ Squamous Cell Carcinoma: A Proposal for Reclassification,” British Journal of Dermatology 156, no. s3 (2007): 8–12, 10.1111/j.1365-2133.2007.07860.x. 17488400

[11] M. T. Fernández‐Figueras , C. Carrato , X. Sáenz , et al., “Actinic Keratosis With Atypical Basal Cells (AK I) is the Most Common Lesion Associated With Invasive Squamous Cell Carcinoma of the Skin,” Journal of the European Academy of Dermatology and Venereology 29, no. 5 (2015): 991–997, 10.1111/jdv.12848. 25428612

[12] M. T. Fernandez Figueras , “From Actinic Keratosis to Squamous Cell Carcinoma: Pathophysiology Revisited,” Journal of the European Academy of Dermatology and Venereology 31, no. S2 (2017): 5–7, 10.1111/jdv.14151. 28263020

[13] D. B. Eisen , M. M. Asgari , D. D. Bennett , et al., “Guidelines of Care for the Management of Actinic Keratosis: Executive Summary,” Journal of the American Academy of Dermatology 85, no. 4 (2021): 945–955, 10.1016/j.jaad.2021.05.056. 34111497 PMC12255291

[14] T. Steeb , A. Wessely , U. Leiter , L. E. French , C. Berking , and M. V. Heppt , “The More the Better? An Appraisal of Combination Therapies for Actinic Keratosis,” Journal of the European Academy of Dermatology and Venereology 34, no. 4 (2020): 727–732, 10.1111/jdv.15998. 31587385

[15] Y. Gilaberte and M. T. Fernández‐Figueras , “Tirbanibulina: revisión de su mecanismo de acción novedoso y de cómo encaja en el tratamiento de la queratosis actínica,” Actas Dermo‐Sifiliográficas 113, no. 1 (2022): 58–66, 10.1016/j.ad.2021.07.006. 35249711

[16] A. Blauvelt , S. Kempers , E. Lain , et al., “Phase 3 Trials of Tirbanibulin Ointment for Actinic Keratosis,” New England Journal of Medicine 384, no. 6 (2021): 512–520, 10.1056/NEJMoa2024040. 33567191

[17] M. C. Kirchberger , M. Gfesser , M. Erdmann , S. Schliep , C. Berking , and M. V. Heppt , “Tirbanibulin 1% Ointment Significantly Reduces the Actinic Keratosis Area and Severity Index in Patients With Actinic Keratosis: Results From a Real‐World Study,” Journal of Clinical Medicine 12, no. 14 (2023): 4837, 10.3390/jcm12144837. 37510952 PMC10381110

[18] E. Campione , A. Rivieccio , R. Gaeta Shumak , et al., “Preliminary Evidence of Efficacy, Safety, and Treatment Satisfaction With Tirbanibulin 1% Ointment: A Clinical Perspective on Actinic Keratoses,” Pharmaceuticals 16, no. 12 (2023): 1686, 10.3390/ph16121686. 38139813 PMC10748142

[19] M. Mansilla‐Polo , C. Abril‐Pérez , D. Martín‐Torregrosa , et al., “Effectiveness, Safety and Satisfaction of 1% Tirbanibulin Ointment in the Treatment of Actinic Keratoses: A Prospective Study in Real Clinical Practice,” Australasian Journal of Dermatology 64, no. 4 (2023): 560–564, 10.1111/ajd.14151. 37675890

[20] F. Li Pomi , M. Vaccaro , G. Pallio , M. Rottura , N. Irrera , and F. Borgia , “Tirbanibulin 1% Ointment for Actinic Keratosis: Results From a Real‐Life Study,” Medicina (B Aires) 60, no. 2 (2024): 225, 10.3390/medicina60020225. PMC10890708 38399512

[21] G. Nazzaro , A. Carugno , P. Bortoluzzi , et al., “Efficacy and Tolerability of Tirbanibulin 1% Ointment in the Treatment of Cancerization Field: A Real‐Life Italian Multicenter Observational Study of 250 Patients,” International Journal of Dermatology 63, no. 11 (2024): 1566–1574, 10.1111/ijd.17168. 38605473

[22] J. DuBois , T. M. Jones , M. S. Lee , et al., “Pharmacokinetics, Safety, and Tolerability of a Single 5‐Day Treatment of Tirbanibulin Ointment 1% in 100 Cm^2^: A Phase 1 Maximal‐Use Trial in Patients With Actinic Keratosis,” Clinical Pharmacology in Drug Development 13, no. 2 (2024): 208–218, 10.1002/cpdd.1368. 38185925

[23] M. Valenti , M. Bianco , A. Narcisi , A. Costanzo , R. Borroni , and M. Ardigò , “Topical Pharmacological Treatment of Actinic Keratoses: Focus on Tirbanibulin 1% Ointment,” Dermatology Practical & Conceptual 14, no. S1 (2024): e2024145S, 10.5826/dpc.1403S1a145S. 39133636 PMC11566729

[24] F. Martora , P. A. Ascierto , M. Scalvenzi , et al., “Tirbanibulin Ointment to Manage Recurrence of Superficial Basal Cell Carcinoma of the Face: Case Report,” Clinical and Experimental Dermatology 49, no. 2 (2024): 183–185, 10.1093/ced/llad334. 37758501

[25] F. Martora , P. Ascierto , M. Palla , et al., “Tirbanibulin: An Alternative Topical Approach to Manage Superficial Basal Cell Carcinoma. Comment on: ‘Successful Treatment of Field Cancerization on the Dorsum of the Hands With 1% Tirbanibulin Ointment’,” Clinical and Experimental Dermatology 48, no. 12 (2023): 1373–1374, 10.1093/ced/llad271. 37566741

[26] D. Blaya Imbernón , M. Finello , C. Labrandero Hoyos , et al., “Successful Treatment of Bowen Disease With 1% Tirbanibulin Ointment,” Clinical and Experimental Dermatology 48, no. 10 (2023): 1184–1186, 10.1093/ced/llad231. 37427812

[27] A. Y. Moore , S. A. Moore , Q. He , P. Rady , and S. K. Tyring , “Tirbanibulin 1% Ointment Eradicates HPV −16 (+) Vulvar High‐Grade Squamous Intraepithelial Lesion,” Journal of the European Academy of Dermatology and Venereology 36, no. 10 (2022): e784–e785, 10.1111/jdv.18265. 35608184

[28] S. Braasch , P. A. Gerber , and S. A. Braun , “Tirbanibulin 1% Ointment as a Potential Novel Treatment for Anogenital Warts,” Journal of the European Academy of Dermatology and Venereology 36, no. 6 (2022): e468–e470, 10.1111/jdv.17957. 35066938

[29] A. Y. Moore and S. Moore , “Topical Tirbanibulin Eradication of Periungual Squamous Cell Carcinoma,” JAAD Case Reports 14 (2021): 101–103, 10.1016/j.jdcr.2021.06.013. 34307817 PMC8283266

[30] F. Li Pomi , L. Peterle , A. d'Aloja , A. Di Tano , M. Vaccaro , and F. Borgia , “Anti‐Aging Effects of Tirbanibulin 1% Ointment: A Real‐Life Experience,” Dermatologic Therapy (Heidelberg) 14, no. 6 (2024): 1683–1696, 10.1007/s13555-024-01178-0. PMC11169325 38740726

[31] F. Li Pomi , A. d'Aloja , M. Rottura , M. Vaccaro , and F. Borgia , “The Skin‐Lightening Power of Tirbanibulin 1% Ointment,” Dermatologic Therapy (Heidelberg) 15 (2024): 95–110, 10.1007/s13555-024-01310-0. PMC11785872 39614963

